# Author Correction: A mouse brain stereotaxic topographic atlas with isotropic 1-μm resolution

**DOI:** 10.1038/s41586-026-11094-2

**Published:** 2026-09-07

**Authors:** Zhao Feng, Xiangning Li, Yue Luo, Xin Liu, Ben Long, Tao Jiang, Xueyan Jia, Xiaowei Chen, Jie Luo, Xiaokang Chai, Zhen Wang, Miao Ren, Xin Lu, Gang Yao, Mengting Zhao, Yuxin Li, Zhixiang Liu, Hong Ni, Chuhao Dou, Shengda Bao, Shicheng Yang, Zoutao Zhang, Jiandong Zhou, Lingyi Cai, Qi Zhang, Ayizuohere Tudi, Chaozhen Tan, Zhengchao Xu, Siqi Chen, Wenxiang Ding, Wenjuan Shi, Anan Li, Hong-wei Dong, Hui Gong, Qingming Luo

**Affiliations:** 1 State Key Laboratory of Digital Medical Engineering, Key Laboratory of Biomedical Engineering of Hainan Province, School of Biomedical Engineering, Hainan University, Haikou, China; 2 HUST-Suzhou Institute for Brainsmatics, JITRI, Suzhou, China; 3 Britton Chance Center for Biomedical Photonics, MOE Key Laboratory for Biomedical Photonics, Wuhan National Laboratory for Optoelectronics, Huazhong University of Science and Technology, Wuhan, China; 4 Department of Neurobiology, David Geffen School of Medicine at UCLA, University of California Los Angeles, Los Angeles, CA USA

**Keywords:** Computational neuroscience, Data integration, Standards

Correction to: *Nature* https://doi.org/10.1038/s41586-025-09211-8 Published online 2 July 2025

In the version of this article initially published, in Extended Data Fig. 2, there was a typographical error in the figure and legend, where “Exm1-Cre” should have read “Emx1-Cre.” In the Extended Data Fig. 4e Source Data and associated figure, the Dice score for the isocortex region of dataset 210692 was incorrectly transcribed, while in the figure legend, the sample size for the medial habenula was incorrectly stated as “*n* = 19” rather than “*n* = 20”. In the Methods “Whole-head dataset acquisition,” in the text now reading “A skull-brain dataset with a voxel resolution of 0.325 × 0.325 × 1 μm^3^”, the values have been updated from “0.32,” in line with the precise value reported in the Results. The text, figures and Source Data are now amended in the HTML and PDF versions of the article. For comparison, the original Extended Data Fig. 4 is available as Supplementary Information accompanying this amendment.

**Supplementary information** is available in the online version of this amendment.

## Supplementary information

Original Extended Data Fig. 4

