# Supplementary material for: A mouse brain stereotaxic topographic atlas with isotropic 1-μm resolution
Source: Nature. 2025 Jul 2;645(8080):448–56. doi: 10.1038/s41586-025-09211-8 (PMC12422980; doi:10.1038/s41586-025-09211-8)
Supplement: Supplementary file 1 — This file contains Supplementary Discussion and Notes. [file 41586_2025_9211_MOESM1_ESM.pdf]

nature portfolio

https://doi.org/10.1038/s41586-025-09211-8

Supplementary information

A mouse brain stereotaxic topographic atlas with isotropic 1-μm resolution

In the format provided by the authors and unedited

Supplementary Discussion

Innovation and contribution The micron-resolution 3D continuous MOST image dataset used to create STAM enables users to visualize individual cells across the entire mouse brain, providing spatial location and morphological information for all cells. To demonstrate its spatial localization capability, we registered single-neuronal circuits datasets from multiple resources onto STAM, localizing neuron somas, branching points, and terminals to their corresponding brain structures of STAM. Additionally, we analyzed the distribution of each neuron's terminals, their projection patterns, and the projection length in different brain regions.

We carefully considered the practical needs of the neuroscience field. Firstly, to meet the requirements of histology and comparative anatomy research, we established a web service for browsing the distribution of brain structures on canonical anatomical and arbitrary-angle planes online. Furthermore, to address the current demands of research on projection circuits and cell types, we have established an online registration tool compatible with both canonical and non-canonical anatomical orientations of brain slices.

Precision of atlas illustrating The MOST-Nissl dataset comprises 14,000 coronal sections with a 1-μm axial interval. Assuming an estimated average drawing speed of one day per section, the total workload would be around 38 years. Therefore, manually delineating brain structural boundaries on each coronal section is unrealistic and unnecessary. With this consideration, we projected ‘standard coronal sections’ with 20-μm thickness for manual annotation of brain structural boundaries. For special cases where certain brain regions undergo appearance, disappearance, or drastic morphological changes in the axial direction, we reduced the axial spacing to capture their fine three-dimensional morphology, by projecting coronal sections at any desired locations.

There are two reasons we chose to track the morphology and boundaries of each brain

region at 20 μm axial intervals. First, the "standard section" is a projection image with a thickness comparable to existing atlases we referred to during illustrating. Secondly, since the average neuronal diameter is on the order of 10 μm, and the cytoarchitectural texture features arise from clusters of aggregated cell bodies, brain region boundaries are expected to change at a scale of approximately 10 μm between adjacent sections. While continuous boundary changes should be observed at an even finer scale, the 20 μm interval allows for efficient identification of structural transitions without compromising anatomical accuracy. Consequently, we retain the flexibility to selectively observe and delineate brain region boundaries at any desired position while improving the efficiency of atlas construction.

Supplementary Notes

Abbreviations of brain regions and nuclei

Below are the full names of the abbreviations that appear in figures and videos, ordered in alphabet.

A13, Dopaminergic A13 group;
ac, anterior commissure;
ACA, Anterior cingulate area;
ACAd, Anterior cingulate area, dorsal part;
ACAd2/3, Anterior cingulate area, dorsal part, layer 2/3;
ACAd5, Anterior cingulate area, dorsal part, layer 5;
ACAd6a, Anterior cingulate area, dorsal part, layer 6a;
ACAv6b, Anterior cingulate area, ventral part, layer 6b;
ACB, Nucleus accumbens;
ACBv, Nucleus accumbens, ventral part;
act, anterior commissure, temporal limb;
ADP, Anterodorsal preoptic nucleus;
AHN, Anterior hypothalamic nucleus;
AI, Agranular insular area;
AIp, Agranular insular area, posterior part;
AIv, Agranular insular area, ventral part;
alv, alveus;
AMB, Nucleus ambiguous;
AOBgl, Accessory olfactory bulb, glomerular layer;
AOBgr, Accessory olfactory bulb, granular layer;
AOBmi, Accessory olfactory bulb, mitral layer;
AON, Anterior olfactory nucleus;

APN, Anterior pretectal nucleus;
ARH, Arcuate hypothalamic nucleus;
AUD, Auditory areas;
AUDd, Dorsal auditory area;
AUDp, Primary auditory area;
AUDpo, Posterior auditory area;
BMA, Basomedial amygdalar nucleus;
BMAa, Basomedial amygdalar nucleus, anterior part
BMAp, Basomedial amygdalar nucleus, posterior part
BLA, Basolateral amygdalar nucleus;
BLAa, Basolateral amygdalar nucleus, anterior part;
BLAp, Basolateral amygdalar nucleus, posterior part;
BLAv, Basolateral amygdalar nucleus, ventral part;
BS, Brainstem;
BST, Bed nucleus of stria terminalis;
CA, Ammon's horn;
CA1, Field CA1 of hippocampus;
CA1d, Field CA1 of hippocampus, dorsal domain;
CA1i, Field CA1 of hippocampus, intermediate domain;
CA1slmd, Field CA1 of hippocampus, stratum lacunosum-moleculare, dorsal domain;
CA1sod, Field CA1 of hippocampus, stratum oriens, dorsal domain;
CA1spd, Field CA1 of hippocampus, pyramidal layer, dorsal domain;
CA1srd, Field CA1 of hippocampus, stratum radiatum, dorsal domain;
CA1v, Field CA1 of hippocampus, ventral domain;
CA2, Field CA2 of hippocampus;
CA2so, Field CA2 of hippocampus, stratum oriens;
CA2sp, Field CA2 of hippocampus, pyramidal layer;
CA2sr, Field CA2 of hippocampus, stratum radiatum;
CA3, Field CA3 of hippocampus;
CA3d, Field CA3 of hippocampus, dorsal domain;
CA3i, Field CA3 of hippocampus, intermediate domain;
CA3sod, Field CA3 of hippocampus, stratum oriens, dorsal domain;
CA3soi, Field CA3 of hippocampus, stratum oriens, intermediate domain;
CA3spdd, Field CA3 of hippocampus, pyramidal layer, rostral-dorsal tip;
CA3spi, Field CA3 of hippocampus, pyramidal layer, intermediate domain;
CA3srd, Field CA3 of hippocampus, stratum radiatum, dorsal domain;
CA3sri, Field CA3 of hippocampus, stratum radiatum, intermediate domain;
CB, Cerebellum;
cbf, cerebellum related fiber tracts;
cc, corpus callosum;
CEA, Central amygdalar nucleus;
CH, Cerebrum;
CL, Central lateral nucleus of the thalamus;
CNU, Cerebral nuclei;

COA, Cortical amygdalar area;
COAa, Cortical amygdalar area, anterior part;
COApl, Cortical amygdalar area, posterior part, lateral zone;
COApm, Cortical amygdalar area, posterior part, medial zone;
CP, Caudoputamen;
cpd, cerebral peduncle;
CSl, Superior central nucleus raphe, lateral part;
CSm, Superior central nucleus raphe, medial part;
cst, corticospinal tract;
CTX, Cerebral cortex;
CTXpl, Cortical plate;
CTXsp, Cortical subplate;
df, dorsal fornix;
DG, Dentate gyrus;
DGcr, crest of the dentate gyrus;
DGd, Dentate gyrus, dorsal part;
DGi, Dentate gyrus, intermediate domain;
DGlb, Dentate gyrus, lateral blade;
DGmb, Dentate gyrus, medial blade;
DGmo, Dentate gyrus, molecular layer;
DGmod, Dentate gyrus, molecular layer, dorsal domain;
DGpo, Dentate gyrus, polymorph layer;
DGpod, Dentate gyrus, polymorph layer, dorsal domain;
DGsg, Dentate gyrus, granular cell layer;
DGsgd, Dentate gyrus, granular cell layer, dorsal domain;
DGsgz, Dentate gyrus, subgranular zone;
DGsod, Dentate gyrus, stratum oriens, dorsal domain;
DGv, Dentate gyrus, ventral domain;
DMH, Dorsomedial nucleus of the hypothalamus;
DMHa, Dorsomedial nucleus of the hypothalamus, anterior part;
DP, Dorsal peduncular area;
DP2/3, Dorsal peduncular area, layer 2/3;
DP5, Dorsal peduncular area, layer 5;
DR, Dorsal nucleus raphe;
ECT, Ectorhinal area;
ENTl, Entorhinal area, lateral part;
EPd, Endopiriform nucleus, dorsal part;
eps, extrapyramidal fiber systems;
EPv, Endopiriform nucleus, ventral part;
FC, fasciola cinerea;
fi, fimbria;
fr, fasciculus retroflexus;
FRP, Frontal pole;
FRP1, Frontal pole, layer 1;

fx, fornix system;
GP, Globus pallidus;
GPe, Globus pallidus, external segment;
GPi, Globus pallidus, internal segment;
GRN, Gigantocellular reticular nucleus;
GU, Gustatory areas;
HIP, Hippocampal region;
HPF, Hippocampal formation;
HY, Hypothalamus;
IB, Interbrain;
icp, inferior cerebellar peduncle;
IG, Induseum griseum;
ILA, Infralimbic area;
int, internal capsule;
isl/OTisl, islands of Calleja;
islm, major island of Calleja;
ISO, isocortex;
LA, Lateral amygdalar nucleus;
LD, Lateral dorsal nucleus of thalamus;
lfbs, lateral forebrain bundle system;
lfbst, lateral forebrain bundle system, thalamus related;
LH, Lateral habenula;
LHA, Lateral hypothalamic area;
LP, Lateral posterior nucleus of the thalamus;
LPO, Lateral preoptic area;
LS, Lateral septal nucleus;
LSc, Lateral septal nucleus, caudal part;
LSr, Lateral septal nucleus, rostral part;
LV, Lateral ventricle;
MA, Magnocellular nucleus;
MB, Midbrain;
mcp, middle cerebellar peduncle;
MD, Mediodorsal nucleus of the thalamus;
MDRNv, Medullary reticular nucleus, ventral part;
MEA, Medial amygdalar nucleus;
MEAad, Medial amygdalar nucleus, anterodorsal part;
MEAav, Medial amygdalar nucleus, anteroventral part;
mfbs, medial forebrain bundle system;
MG, Medial geniculate complex;
MGv, Medial geniculate complex, ventral part;
MH, Medial habenula;
ml, medial lemniscus;
MO, Somatomotor areas;
MOp, Primary motor area;

MOp1, Primary motor area, layer 1;
MOp2/3, Primary motor area, layer 2/3;
MOp5, Primary motor area, layer 5;
MOs, Secondary motor area;
MOs2/3, Secondary motor area, layer 2/3;
MOs5, Secondary motor area, layer 5;
MOs6a, Secondary motor area, layer 6a;
MPN, Medial preoptic nucleus;
MPO, Medial preoptic area;
MPT, Medial pretectal area;
MRN, Midbrain reticular nucleus;
MS, Medial septal nucleus;
mtt, mammillothalamic tract;
MY, medulla;
NB, Nucleus of the brachium of the inferior colliculus;
NDB, Diagonal band nucleus;
NLL, Nucleus of the lateral lemniscus;
NLLv, Nucleus of the lateral lemniscus, ventral part;
NOT, Nucleus of the optic tract;
NPC, Nucleus of the posterior commissure;
och, optic chiasm;
OLF, Olfactory areas;
OP, Olivary pretectal nucleus;
opt, optic tract;
OT, Olfactory tubercle;
OV, Vascular organ of the lamina terminalis;
P, Pons;
PA, Posterior amygdalar nucleus;
PAA, Piriform-amygdalar area;
PAG, Periaqueductal gray;
PAL, Pallidum;
pc, posterior commissure;
PD, Posterodorsal preoptic nucleus;
PeF, Perifornical nucleus;
PERI, Perirhinal area;
PG, Pontine gray;
PIR, Piriform area;
PL, Prelimbic area;
PL5, Prelimbic area, layer 5;
PO, Posterior complex of the thalamus;
PPN, Pedunculopontine nucleus;
PPT, Posterior pretectal nucleus;
PRE3, Presubiculum, layer 3;
PRNr, Pontine reticular nucleus;

ProSUB, Prosubiculum;
PS, Parastrial nucleus;
PTLp, Posterior parietal association areas;
PVi, Periventricular hypothalamic nucleus, intermediate part;
PVH, Paraventricular hypothalamic nucleus;
PVT, Paraventricular nucleus of the thalamus;
py, pyramid;
RHP, Retrohippocampal region;
RM, Nucleus raphe magnus;
RPA, Nucleus raphe pallidus;
RR, Midbrain reticular nucleus, retrorubral area;
RSP, Retrosplenial area;
RSPagl, Retrosplenial area, lateral agranular part;
RSPd, Retrosplenial area, dorsal part;
RSPv, Retrosplenial area, ventral part;
RSPv1, Retrosplenial area, ventral part, layer 1;
RSPv2/3, Retrosplenial area, ventral part, layer 2/3;
RSPv5, Retrosplenial area, ventral part, layer 5;
RT, Reticular nucleus of the thalamus;
SBPV, Subparaventricular zone;
SCm, Superior colliculus, motor related;
scp, superior cerebellar peduncle;
SF, Septofimbrial nucleus;
SI, Substantia innominate;
sm, stria medullaris;
SNr, Substantia nigra, reticular part;
SPFp, Subparafascicular nucleus, parvicellular part;
SPVI, Spinal nucleus of the trigeminal, interpolar part;
SS, Somatosensory areas;
SSp, Primary somatosensory area;
SSp-bfd, Primary somatosensory area, barrel field;
SSp-ll, Primary somatosensory area, lower limb;
SSp-ul, Primary somatosensory area, upper limb;
SSs, Supplemental somatosensory area;
st, stria terminalis;
STN, Subthalamic nucleus;
STR, Striatum;
SUB, Subiculum;
SUBdm, Subiculum, dorsal part, molecular layer;
SUBdsp1, Subiculum, dorsal part, pyramidal layer, layer 1;
SUBdsp4, Subiculum, dorsal part, pyramidal layer, layer 4;
SUBdsr, Subiculum, dorsal part, stratum radiatum;
SUBvm, Subiculum, ventral part, molecular layer;
SUBvsp, Subiculum, ventral part, pyramidal layer;

SUBvsp1, Subiculum, ventral part, pyramidal layer, layer 1;
SUBvsp4, Subiculum, ventral part, pyramidal layer, layer 4;
SUBvsr, Subiculum, ventral part, stratum radiatum;
SUMm, Supramammillary nucleus, medial part;
TEa, Temporal association areas;
TH, Thalamus;
TRS, Triangular nucleus of septum;
TT, Taenia tecta;
TT2, Taenia tecta, layer 2;
TTd, Taenia tecta, dorsal part;
TTd1, Taenia tecta, dorsal part, layer 1;
TTd2, Taenia tecta, dorsal part, layer 2;
TU, Tuberal nucleus;
V3, third ventricle;
VAL, Ventral anterior-lateral complex of the thalamus;
vhc, ventral hippocampal commissure;
VIIn, facial nerve;
VIS, Visual areas;
VISal, Anterolateral visual area;
VISam, Anteromedial visual area;
VISp, Primary visual area;
VISpm, Posteromedial visual area;
VLPO, Ventrolateral preoptic nucleus;
VM, Ventral medial nucleus of the thalamus;
VMH, Ventromedial hypothalamic nucleus;
VP, Ventral posterior complex of the thalamus;
VPL, Ventral posterolateral nucleus of the thalamus;
VPM, Ventral posteromedial nucleus of the thalamus;
VS, ventricular systems;
ZI, Zona incerta;
ZIr, Zona incerta, rostral part.
