# Supplementary material for: A mouse brain stereotaxic topographic atlas with isotropic 1-μm resolution
Source: Nature. 2025 Jul 2;645(8080):448–56. doi: 10.1038/s41586-025-09211-8 (PMC12422980; doi:10.1038/s41586-025-09211-8)
Supplement: Supplementary file 2 — Reporting Summary [file 41586_2025_9211_MOESM2_ESM.pdf]

nature portfolio

Corresponding author(s): Hongwei Dong, Hui Gong, Qingming Luo

Last updated by author(s): May 18, 2025

Reporting Summary

Nature Portfolio wishes to improve the reproducibility of the work that we publish. This form provides structure for consistency and transparency in reporting. For further information on Nature Portfolio policies, see our Editorial Policies and the Editorial Policy Checklist.

Statistics

For all statistical analyses, confirm that the following items are present in the figure legend, table legend, main text, or Methods section.

n/a	Confirmed	
☐	☒	The exact sample size (*n*) for each experimental group/condition, given as a discrete number and unit of measurement
☐	☒	A statement on whether measurements were taken from distinct samples or whether the same sample was measured repeatedly
☒	☐	The statistical test(s) used AND whether they are one- or two-sided<br>*Only common tests should be described solely by name; describe more complex techniques in the Methods section.*
☒	☐	A description of all covariates tested
☒	☐	A description of any assumptions or corrections, such as tests of normality and adjustment for multiple comparisons
☐	☒	A full description of the statistical parameters including central tendency (e.g. means) or other basic estimates (e.g. regression coefficient) AND variation (e.g. standard deviation) or associated estimates of uncertainty (e.g. confidence intervals)
☒	☐	For null hypothesis testing, the test statistic (e.g. *F*, *t*, *r*) with confidence intervals, effect sizes, degrees of freedom and *P* value noted<br>*Give P values as exact values whenever suitable.*
☒	☐	For Bayesian analysis, information on the choice of priors and Markov chain Monte Carlo settings
☒	☐	For hierarchical and complex designs, identification of the appropriate level for tests and full reporting of outcomes
☒	☐	Estimates of effect sizes (e.g. Cohen's *d*, Pearson's *r*), indicating how they were calculated

Our web collection on statistics for biologists contains articles on many of the points above.

Software and code

Policy information about availability of computer code

Data collection	No software for collecting data is used for this study.
Data analysis	The ImageJ (v 1.53k) is available at https://imagej.net/ij/download.html.<br>The ANTs tool (v 2.1.0) is available at https://github.com/ANTsX/ANTs.<br>The BrainsMapi tool (No version number available) is available upon request.<br>The Visualization Toolkit (v 9.3.0) is available at https://github.com/Kitware/VTK.<br>The Blender software (v 4.3) is available at https://www.blender.org/download/.<br>The Three.js (v 0.157.0) is available at https://github.com/mrdoob/three.js.<br>The Zoomify tool (Enterprise Developer 4) we purchased is no longer maintained by the producer https://zoomify.com/.<br>The neuroglancer framework (v 2.29) is available at https://github.com/google/neuroglancer.<br>The Matlab software (v 2023b) is a commercial production, and can be purchased at https://www.mathworks.com/products/matlab.html.<br>The Amira Software (v 6.1.1) is a commercial software, and can be purchased through https://www.thermofisher.com/.<br>The SVRnet package (No version number available) is available at https://github.com/farrell236/SVRnet.

For manuscripts utilizing custom algorithms or software that are central to the research but not yet described in published literature, software must be made available to editors and reviewers. We strongly encourage code deposition in a community repository (e.g. GitHub). See the Nature Portfolio guidelines for submitting code & software for further information.

Data

Policy information about availability of data

All manuscripts must include a data availability statement. This statement should provide the following information, where applicable:

- Accession codes, unique identifiers, or web links for publicly available datasets
- A description of any restrictions on data availability
- For clinical datasets or third party data, please ensure that the statement adheres to our policy

The 1 µm resolution MOST-Nissl dataset used to construct STAM, the labeling images, and vectorized boundaries of brain structures, are available through https://atlas.brainsmatics.cn/STAM/. Readers can browse this link and find the desired way to navigate or download our data, or query Supplementary Table 3 to visit the specific gene-type neuron distribution datasets used for validating STAM, the comparison of the MOST-Nissl dataset with the Nissl-staining sections from brainmaps.org, and more results for Extended Data Fig. 5, Extended Data Fig. 7, and Extended Data Fig. 10. The ARA and CCF referred in this study can be accessed by https://atlas.brain-map.org. The WHS data were downloaded from https://www.nitrc.org/projects/incfwhsmouse. The brain region labels of MBSC used in our coronal plane visualization service are obtained through https://datadryad.org/dataset/doi:10.5061/dryad.t1g1jwsxw. The ISH image data from Allen Institute used in this study can be accessed by https://mouse.brain-map.org/. The neuron morphology data used by STAM's neuronal connectivity web service include datasets from Brain Image Library (https://www.brainimagelibrary.org/), under the following BIL ID, which is used as the identifier to query dataset at https://api.brainimagelibrary.org/web/: ace-ban-out, ace-ban-owl, ace-ban-own, ace-ban-pad, ace-ban-pal, ace-ban-pan, ace-ban-pay, ace-ban-pen, ace-ban-pet, ace-ban-pie, ace-ban-pig, war, wax, wet, ace-die-age, ace-ban-rig, who, ace-did-who, ace-add-vat, ace-add-vex, ace-ban-pot, ace-add-wag, ace-ban-pry, ace-ban-pun, ace-add-was, ace-ban-put, ace-add-web, ace-ban-ran, ace-ban-rat, ace-ban-raw, ace-ban-red, ace-ban-rid, win, wit, zoo, all, ace-zip, ace-ace, ace-act, ace-add, ace-age, ace-aim, ace-air, ace-and, ace-ant, ace-ape, ace-arm, ace-art, ace-ash, ace-ask, ace-ban-rip, ace-die-ant, ace-did-win, ace-ban, ace-bat, ace-bay, ace-bed, ace-bet, ace-bid, ace-big, ace-bin, ace-bit, ace-bog, ace-boo, ace-box, ace-bug, ace-bun, ace-bus, ace-cab.

Research involving human participants, their data, or biological material

Policy information about studies with human participants or human data. See also policy information about sex, gender (identity/presentation), and sexual orientation and race, ethnicity and racism.

Reporting on sex and gender	Not applicable
Reporting on race, ethnicity, or other socially relevant groupings	Not applicable
Population characteristics	Not applicable
Recruitment	Not applicable
Ethics oversight	Not applicable

Note that full information on the approval of the study protocol must also be provided in the manuscript.

Field-specific reporting

Please select the one below that is the best fit for your research. If you are not sure, read the appropriate sections before making your selection.

☒ Life sciences ☐ Behavioural & social sciences ☐ Ecological, evolutionary & environmental sciences

For a reference copy of the document with all sections, see nature.com/documents/nr-reporting-summary-flat.pdf

Life sciences study design

All studies must disclose on these points even when the disclosure is negative.

Sample size	No formal statistical method was used to predetermine sample size. The sample sizes were chosen based on established practices in atlas development, where a single, carefully prepared sample is used as the 'basis' to ensure consistent cytoarchitecture information, assisted by auxilary image modalities to supplement extra details for structure delineation. Specifically, 3 adult C57BL/6J mice were used: one for generating the whole-brain Nissl-stained image dataset for atlas construction (the 'basis' sample), one for immunohistochemical labeling, and one for obtaining the whole-head sample used to define cranial landmarks. In addition, 22 transgenic mice were used to obtain specific neuronal distribution patterns. These sample sizes are considered sufficient and appropriate for the structural delineation purpose, as this study focuses on the construction of a brain atlas, which typically relies on high-quality representative samples rather than group comparisons or hypothesis testing.
Data exclusions	No data were excluded from the analyses.
Replication	This study focuses on the construction of a high-resolution mouse brain atlas based on Nissl-stained cytoarchitectural imaging and is supported by various auxiliary imaging modalities. Given the structural and anatomical nature of the data, the findings are not subject to experimental variability in the traditional sense. The protocol of this study follows established practices in stereotaxic atlas development, therefore, we confirm no findings were identified that could not be reproduced.

Randomization	This study does not involve allocation of animals into experimental groups, as it focuses on brain atlas construction based on high-resolution structural imaging. All samples were used for specific and predetermined purposes (e.g., one for Nissl-stained imaging, one for immunohistochemistry, one for intact head imaging, and 22 transgenic mice for specific neuron labeling). Therefore, randomization and control of covariates were not applicable in this context.
Blinding	Blinding was not relevant to this study. The research did not involve group allocation, treatment comparisons, or outcome assessments subject to observer bias. All imaging data were acquired and analyzed based on predetermined anatomical and structural criteria for brain atlas construction, ensuring objectivity in data interpretation.

Reporting for specific materials, systems and methods

We require information from authors about some types of materials, experimental systems and methods used in many studies. Here, indicate whether each material, system or method listed is relevant to your study. If you are not sure if a list item applies to your research, read the appropriate section before selecting a response.

Materials & experimental systems

n/a	Involved in the study
☐	☒ Antibodies
☒	☐ Eukaryotic cell lines
☒	☐ Palaeontology and archaeology
☐	☒ Animals and other organisms
☒	☐ Clinical data
☒	☐ Dual use research of concern
☒	☐ Plants

Methods

n/a	Involved in the study
☒	☐ ChIP-seq
☒	☐ Flow cytometry
☒	☐ MRI-based neuroimaging

Antibodies

Antibodies used	For the immunohistochemistry, the mouse brain was sectioned at 70 μm thickness by a vibratome (Leica, VS1200S). the slices were washed with PBS, blocked with 5% bovine serum albumin, and then incubated with the primary antibodies (anti- NeuN, mouse, Covance, SIG-39860, 1:1000 dillution; anti- NF160, rabbit, Abcam, ab64300, 1:1000 dillution) overnight at 4 °C. After washing with PBS, the secondary antibodies, namely Alexa Fluor 488 goat anti-mouse immunoglobulin G (IgG) (Invitrogen, Carlsbad, CA, United States, A11029, 1:1,000 dilution), and Alexa Fluor 594 goat anti-rabbit IgG (Invitrogen, Carlsbad, CA, United States, A11037, 1:1000 dilution) were applied for 2 hours at room temperature. Imaging was done afterwards with a multichannel fluorescence slide microscope (Olympus VS120, Tokyo, Japan).
Validation	These are all well characterized commercial antibodies.The specificity of the primary and secondary antibodies was validated by the manufacturers.<br>anti-NeuN: https://www.antibodypedia.com/gene/32645/RBFOX3/antibody/1457501/SIG-39860<br>anti-NF160: https://www.labome.com/product/Abcam/ab64300.html

Animals and other research organisms

Policy information about studies involving animals; ARRIVE guidelines recommended for reporting animal research, and Sex and Gender in Research

Laboratory animals	3 C57BL/6J mice were used, 1 for Nissl staining, 1 for immunohistochemistry, 1 for acquiring the whole head sample. Another 22 transgenetic mice were used for acquiring specific gene-type neuron distribution images. The C57BL/6J mice were all 8-week old, while the age of 22 transgenetic mice varies from 6-week to 36-week. The details can be queried in the Supplementary Table 2.
Wild animals	The study did not involve wild animals.
Reporting on sex	The findings apply to both male and female mice. The information about sex is reported in Supplementary Table 2. For the 3 mice used to construct our atlas, the sex is male. For the mouse samples used to collect the neuron distribution datasets with specific gene types, there are 8 male samples and 14 female samples.
Field-collected samples	The study did not involve sample collected from the fields.
Ethics oversight	the Institutional Animal Ethics Committee of HUST-Suzhou Institute for Brainsmatics

Note that full information on the approval of the study protocol must also be provided in the manuscript.

Plants

Seed stocks	Not applicable
Novel plant genotypes	Not applicable
Authentication	Not applicable
