# Supplementary material for: Author Correction: A mouse brain stereotaxic topographic atlas with isotropic 1-μm resolution
Source: Nature. 2026 Sep 7;657(8133):E19. doi: 10.1038/s41586-026-11094-2 (PMC13600918; doi:10.1038/s41586-026-11094-2)
Supplement: Supplementary file 1 [file 41586_2026_11094_MOESM1_ESM.pdf]

nature portfolio

https://doi.org/10.1038/s41586-026-11094-2

# Supplementary information

# Author Correction: A mouse brain stereotaxic topographic atlas with isotropic 1-μm resolution

In the format provided by the authors and unedited

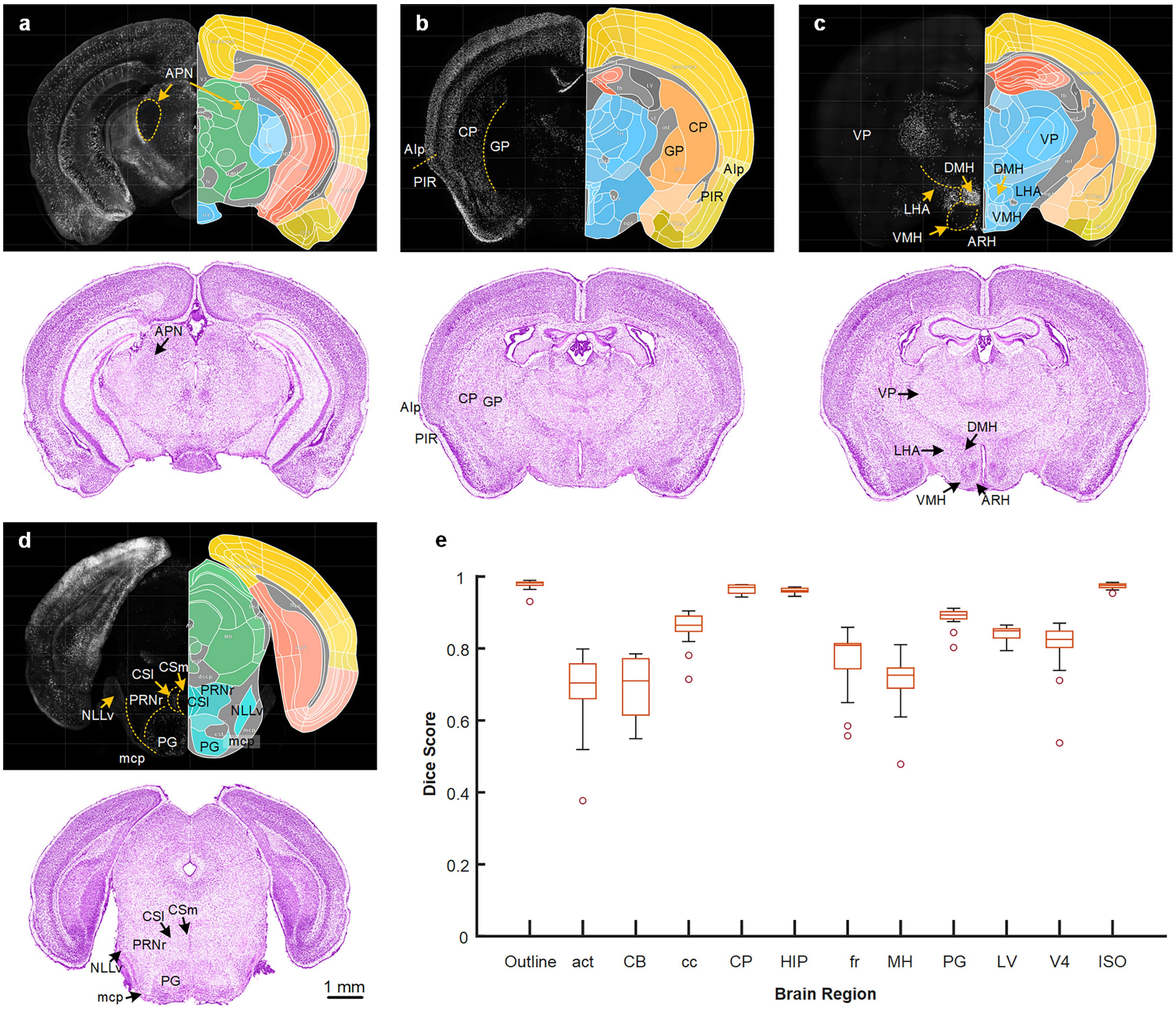


**Extended Data Fig. 4 | Specific gene-type neuron distributions aid structural delineation. a-d**, Representative coronal planes from SOM-Cre, Camk2-Cre, GAL-Cre and CRH-Cre datasets respectively. In each, the left half of the first row shows structure boundaries (yellow dashed lines) inferred from neuron distribution patterns, and the right half shows the corresponding STAM atlas delineation. The images on the second row are Nissl-stained sections at the same location, with the black texts as the migrated brain region identifications from the corresponding specific neuron distribution images. The left half image shown in each panel is the representative coronal images from one specific neuron distribution dataset. Scale bar applies to (**a-d**) and is located below (**d**). **e**, Quantitative evaluation of registration accuracy. Dice scores are shown as box plots for each brain region (X-axis), with Y-axis displaying the interquartile range (orange box), median (orange line), minimum/maximum values (whiskers), and outliers (orange circles). Note that not all brain regions shown in (**e**) are identifiable in all specific gene-type neuron distribution dataset (n, number of biologically independent datasets for evaluating the accuracy of registering certain brain structure. n = 22 for Outline, CB, cc, HIP, PG, LV and V4; n = 20 for act; n = 19 for fr and MH; n = 13 for ISO; n = 12 for CP).
